# Neuropathologie der Demenzen

**DOI:** 10.1007/s10354-021-00848-4

**Published:** 2021-06-15

**Authors:** Sigrid Klotz, Ellen Gelpi

**Affiliations:** 1 Abteilung für Neuropathologie und Neurochemie, Universitätsklinik für Neurologie, Medizinischer Universitätscampus Wien, Ebene 4J, Währinger Gürtel 18–20, 1090 Wien, Österreich; 2 Österreichisches Referenzzentrum zur Erfassung und Dokumentation menschlicher Prionen-Erkrankungen (ÖRPE), Wien, Österreich

**Keywords:** Demenz, Neuropathologie, Neurodegeneration, Biomarker, Proteinopathien, Dementia, Neuropathology, Neurodegeneration, Biomarker, Proteinopathies

## Abstract

Demenz ist die klinische Folge verschiedener neurologischer Erkrankungen mit einer Vielzahl von Ätiologien. Dabei ist die genaue Kenntnis der zugrunde liegenden pathologischen Veränderungen entscheidend für die passgenaue Versorgung der Patienten und für die Entwicklung geeigneter Krankheitsbiomarker. Eine definitive Diagnose vieler dieser Erkrankungen, insbesondere der neurodegenerativen Formen, kann nur nach gründlicher postmortaler neuropathologischer Untersuchung gestellt werden. Dies unterstreicht die Wichtigkeit der Durchführung einer Gehirnautopsie und die Relevanz einer engen Zusammenarbeit zwischen Klinikern, Neuroradiologen und Neuropathologen sowie mit Grundlagenforschern. Ziel der vorliegenden Arbeit ist es, einen kurzen Überblick über die Neuropathologie der Demenz mit Schwerpunkt auf neurodegenerative Erkrankungen zu geben, um die interdisziplinäre Zusammenarbeit weiter zu fördern.

## Abstract (en)

Dementia is the clinical consequence of various neurological disorders with a multitude of etiologies. Precise knowledge of the underlying pathologies is essential for an accurate treatment of patients and for the development of suitable disease biomarkers. A definite diagnosis of many of the disorders, particularly for neurodegenerative ones, can only be made after a thorough postmortem neuropathological examination. This highlights the importance of performing a brain autopsy and the relevance of a close interaction between clinicians, neuroimaging disciplines and neuropathologists as well as with basic researchers. This article aims to give a brief overview on the neuropathology of dementia focusing on neurodegenerative diseases, to further facilitate interdisciplinary collaboration.

Der Terminus Demenz ist eine klinische Konsequenz von unterschiedlichsten neurologischen Erkrankungen, sodass die genaue Kenntnis der potenziell zugrunde liegenden Ursache für eine optimale Versorgung unumgänglich ist. Trotz bedeutsamer und richtungsweisender Weiterentwicklungen im Bereich der klinischen Diagnostik mittels bildgebender Methoden und diverser Blut- und liquorbasierter Biomarker kann bis dato zumeist keine definitive Diagnose der meisten demenziellen, zumindest der neurodegenerativen Erkrankungsformen erfolgen. Der neuropathologischen Autopsie fällt daher ein besonderer Stellenwert zu, wenngleich diese erst nach dem Ableben und zumeist nach fortgeschrittener Erkrankung durchgeführt wird. Eine definitive Diagnose ist nicht zuletzt auch im Hinblick auf eine potenzielle hereditäre Komponente von Bedeutung. Die Autopsie stellt auch eine Schnittstelle zwischen klinischer und Grundlagenforschung dar, die auch von wesentlicher Bedeutung angesichts künftiger zielgerichteter Therapien ist. Eine intensive Zusammenarbeit der unterschiedlichen beteiligten Disziplinen ist daher ausschlaggebend für eine fruchtbare neurowissenschaftliche Forschung.

Grundlage der klinischen Symptome sind die neuropathologischen Veränderungen, die zu neuronaler bzw. synaptischer Dysfunktion in bestimmten Hirnregionen und in bestimmten Netzwerken führen. Hier können zum einen die Verteilung innerhalb neuroanatomischer Regionen und andererseits die molekularbiologische Basis mit Untersuchung von unterschiedlichen Zellelementen sowie Zellkompartimenten und -funktionen unterschieden werden. Die Ursachen dieser neuronalen Dysfunktion, die letztlich zu einer Demenz führen kann, sind breit. Als grobe Unterteilung können die primär neurodegenerativen Ursachen von nicht primär neurodegenerativen Ursachen unterschieden werden.

## Primär neurodegenerative Ursachen

### Allgemeines Konzept.

Neurodegeneration beruht auf dem progredienten Nervenzellverlust eines zuvor regulär funktionierenden Gehirns, begleitet von einer chronischen reaktiv proliferativen Gliose und einer Mikrogliaaktivierung. Die topografische Verteilung bestimmt zumeist den klinischen Phänotyp, die zellulären Veränderungen erlauben die frühe Identifizierung pathologischer Prozesse (präklinisch) sowie eine molekulare Klassifikation der unterschiedlichen Erkrankungen. Diese stellen zum Teil die Basis für die Entwicklung von Biomarkern und neuen Therapien dar.

### Proteinopathiekonzept.

Die primär neurodegenerativen Demenzen sind charakterisiert durch eine progrediente Ablagerung von pathologischen Proteinen. Zumeist entstehen diese pathologischen Proteine durch Fehlfaltung eines physiologisch vorhandenen Proteins [1]. Aus bisher noch ungeklärten Gründen kommt es zu einer Konformationsänderung mit Ausbildung von Oligo- und Multimeren, welche sich schließlich teilweise zu unlöslichen Fibrillen aneinanderlegen und deren Fehlkonformation an weitere zuvor „gesunde“ Proteine übertragen [2]. Diese Fehlfaltung führt zu einem Funktionsverlust, oft beginnend an den Synapsen, und in Folge zu einem Nervenzellverlust.

### Klassifikation.

Die meisten neurodegenerativen Erkrankungen können anhand der Art, der Lokalisation sowie der Morphologie der abnormen Proteindeposite klassifiziert werden (siehe Abb. 2) [3]. Die häufigsten vorkommenden Proteinablagerungen stellen Tau, beta-Amyloid, alpha-Synuclein und TDP-43(„transactive response DNA binding protein 43 kDa“)-Proteine dar. Die Einteilung anhand dieser pathologischen Proteinablagerungen hat die Begriffe der Tauopathie bzw. Tau-Proteinopathie, Synucleinopathie bzw. Synuclein-Proteinopathie und TDP43-Proteinopathie hervorgebracht. Allerdings könnte eine solche Einteilung etwas zu vereinfachend sein, da Kombinationen mehrerer Pathologien nicht selten sind [4].

Die große Gruppe der sog. „Tauopathien“ kann zudem weiter anhand unterschiedlicher Tau-Protein-Isoformen, welche durch alternatives Splicing entstehen, eingeteilt werden [5]. Es können hier in Abhängigkeit von der Zahl der „Tandem-Repeats“ im Bereich der Mikrotubulibindungsdomäne des Proteins (3 oder 4), 3‑repeat(3R)- und 4‑repeat(4R)-Tau-Isoformen unterschieden werden und die Pathologien entsprechend als 3R-, 4R- oder gemischte 3R + 4R-Tauopathie klassifiziert werden. Auch Beta-Amyloid, welches durch Aktivität von Sekretasen aus dem Vorläuferprotein von Beta-Amyloid („amyloid precursor protein“ [APP]) gebildet wird, kann anhand seiner Molekülgröße weiter unterteilt werden. Es kann einerseits Beta-Amyloid mit einer Moleküllänge von 40 Aminosäuren (Aβ40) sowie Beta-Amyloid mit einer Moleküllänge von 42 Aminosäuren (Aβ42) unterschieden werden.

### Lokalisation.

Bezüglich der Lokalisation der Pathologie werden extra- von intrazellulären Depositen und auch die betroffenen Zellformen unterschieden. Neben Neuronen, die bisher im Fokus der Wissenschaft standen, spielen auch Gliazellen inklusive der Mikroglia mit ihrer Bedeutung in der Homöostase eine bedeutsame Rolle im Bereich der Neurodegeneration [6–8].

### Morphologie und Visualisierung.

Eine weitere Differenzierung der neurodegenerativen Erkrankungen erfolgt anhand der Morphologie der entsprechenden Proteinablagerungen. Diese können oftmals bereits in der histologischen Basisfärbung, der Hämatoxylin-Eosin-Färbung (H&E), als Einschlusskörper identifiziert werden. Zudem können fibrilläre Strukturen mithilfe von Versilberungstechniken wie der Bielschowsky- oder Gallyas-Silberfärbung im histologischen Präparat visualisiert werden. Eine weitere, besonders sensitive Methode zur Visualisierung von pathologischen Proteinablagerungen an Gewebeschnitten stellen immunhistochemische Färbungen dar.

### Selektive Vulnerabilität und „protein spreading“.

Ein grundlegendes Konzept der neurodegenerativen Erkrankungen ist das der selektiven Vulnerabilität. Hierbei hat sich gezeigt, dass pathologische Proteinablagerungen sich in bestimmten Zellen in bestimmten neuroanatomischen Region ablagern bzw. dass bestimmte Arten von Neuronen eine Vulnerabilität für bestimmte Proteinopathien aufweisen [9]. Ausgehend von diesem primär involvierten Bereich, kommt es zu einer progredienten Ausbreitung der Pathologie. Diese Ausbreitung beruht auf dem sog. Protein-spreading-Prinzip. Dies basiert darauf, dass die pathologische Konformationsänderung der verschiedenen Proteinopathien eine gewisse Übertragbarkeit und Induktion einer Konformationsänderung in „gesunden“ Proteinen aufweist [10]. Diese Übertragbarkeit einer pathologischen Konformation eines Proteins wurde erstmals im Kontext von Prionenerkrankungen beschrieben, weshalb auch der Begriff „prion-like spreading“ verwendet wird [11]. Hierbei kommt den Prionenerkrankungen jedoch eine Sonderstellung zu, da die Übertragbarkeit von Prionen nicht nur von Zelle zu Zelle, sondern unter bestimmten Bedingungen auch in vivo von Organismus zu Organismus gegeben ist. Eine solche Übertragbarkeit ist für andere Proteinopathien zwar im experimentell-wissenschaftlichen Kontext vorhanden [12–14], jedoch scheint dies in vivo zumeist eine weniger bedeutsame Rolle zu spielen, wenngleich dies noch nicht gänzlich geklärt ist und weiterer Forschung bedarf [15].

### Translation Neuropathologie – Biomarker – klinische Forschung.

Die progrediente, stereotype Ausbreitung von pathologischen Ablagerungen entlang bestimmter neuronaler Netzwerke dient als Grundlage für neuropathologische Einteilung in Krankheitsstadien, anhand derer die Ausdehnung der Pathologie genauer beschrieben werden kann und deren Translation in Ante-mortem-Untersuchungen ein Ziel der Biomarkerforschung ist.

Neben der Rolle der Charakterisierung der pathologischen Veränderungen im Kontext der Biomarkerforschung (*Bioflüssigkeiten und Neuroimaging*) stellt diese auch eine Basis für potenzielle zielgerichtete Therapien dar. Neben Antikörpertherapien gegen die unterschiedlichen krankheitsassoziierten Proteine (wie etwa die Anti-Tau-Antikörper Gosuranemab und Semorinemab oder die Anti-Beta-Amyloid-Antikörper Solanezumab oder Aducanumab), die seit einiger Zeit untersucht werden [16], stellt die Autophagie ein potenzielles Therapietarget dar [17]. Da die Anhäufung der pathologischen Proteindeposite u. a. auch mit einem fehlenden Abbau über zelleigene „Clearing“-Mechanismen einhergeht, wird die Förderung der Autophagie derzeit als möglicher Therapieansatz untersucht. Ein weiteres potenziell vielversprechendes Therapieziel stellen die Mikroglia und Neuroinflammation dar [18].

## Demenz vom Alzheimer-Typ

Die häufigsten Ursachen eines demenziellen Zustandsbilds sind zugrunde liegende neuropathologische Veränderungen vom Alzheimer-Typ. Die Häufigkeit von solchen Veränderungen nimmt mit zunehmendem Alter zu, weshalb im Anbetracht des demografischen Wandels diese Pathologien an Bedeutung gewinnen [19].

Zuerst beschrieben wurde die Alzheimer-Erkrankung 1906 vom deutschen Psychiater und Neuropathologen Alois Alzheimer [20, 21]. Mithilfe von Silberfärbungen beschrieb er bereits damals intrazelluläre neurofibrilläre Bündel (sog. „neurofibrillary tangles“ [NFT]) und amorphe extrazelluläre Ablagerungen (senile Plaques, neuritische Plaques, bestehend aus Beta-Amyloid). Diese Beobachtungen sind noch heute aktuell, die morphologischen und molekularen Veränderungen wurden mittlerweile genauer charakterisiert [22]. Neurofibrilläre Tangles innerhalb von Nervenzellen bestehen aus hyperphosphoryliertem Tau, einem Mikrotubuli-assoziierten Protein, welches in seiner pathologischen Form filamentöse Strukturen ausbildet („paired helical filaments“). Je nach Form der Nervenzellen, in denen sie sich befinden, bilden diese auch unterschiedlich geformte Strukturen aus (z. B. flammenförmige Tangles in Pyramidenzellen oder globöse Tangles in großen rundlichen cholinergen Nervenzellen). Daneben aggregiert das pathologische Tau auch in Nervenzellfortsätzen und bildet sowohl zarte fadenförmige Tau-positive Inklusionen in Dendriten, sog. Neuropilfäden („neuropil threads“), als auch verdickte dystrophe Neuriten, die zumeist um Beta-Amyloid-Cores entstehen, aus. Es kommt bei der Alzheimer-Pathologie zu einer gemischten Ablagerung von 3R- und 4R-Tau. Die extrazellulären Plaques bestehen aus dichten Ablagerungen von Beta-Amyloid-Protein. Diese Plaques bestehen üblicherweise primär aus Beta-Amyloid mit einer Moleküllänge von 42 Aminosäuren (Aβ42).

**Figure 1:**
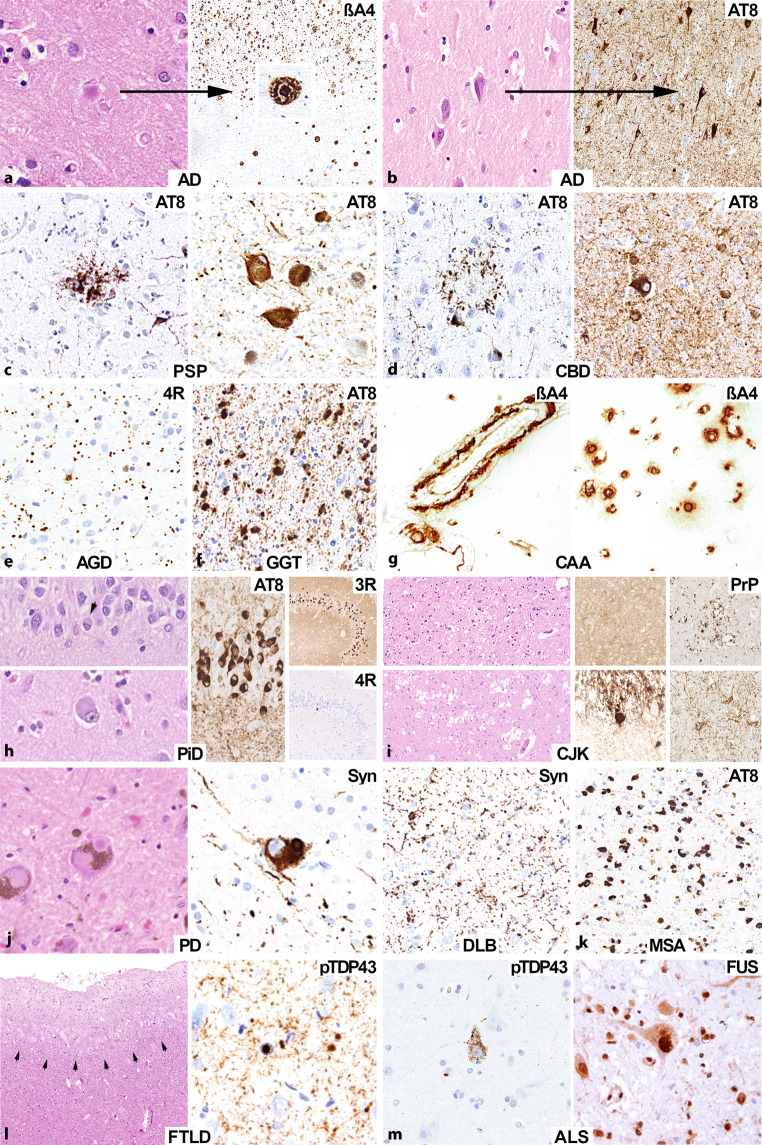

Die Beta-Amyloid-Plaques und NFT sind teilweise bereits in der histologischen Basisfärbung, der Hämatoxylin-Eosin-Färbung (H&E), v. a. aber in traditionellen Silberfärbungen zu sehen und können auch mithilfe von immunhistochemischen Färbungen gegen Beta-Amyloid bzw. gegen hyperphosphoryliertes Tau visualisiert werden (Abb. 1a, b). Immunhistochemisch können zudem Elemente des Ubiquitin-Proteasoms bzw. „Autophagiemarker“ wie p62 eingesetzt werden. Allerdings weisen diese Marker keinerlei Proteinspezifität auf, weshalb sie primär als Screeningwerkzeug pathologischer Einschlüsse angewendet werden [23]. Es können daher heute pathologische Proteindeposite, die in der Vergangenheit lediglich als „Ubiquitin-positiv“ beschrieben werden konnten, gezielt und spezifisch nachgewiesen werden.

**Figure 2:**
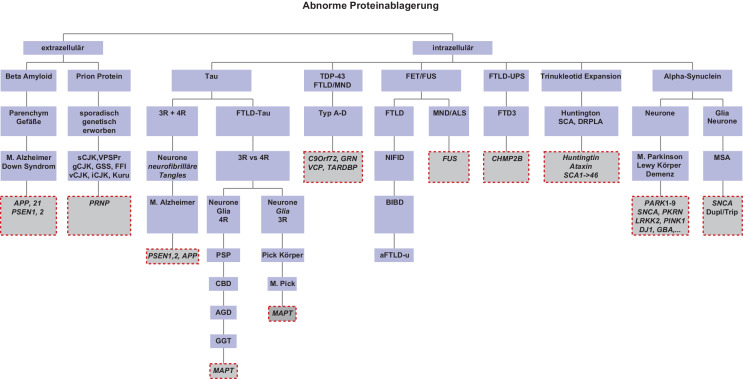

Mit zunehmender Dauer der Erkrankung kommt es zu einer stereotypen Ausbreitung der pathologischen Veränderungen. Es konnten Heiko Braak et al. 6 Stadien der NFT-Pathologie mit 2 transentorhinalen Stadien (I–II), 2 limbischen Stadien (III–IV) und 2 isokortikalen Stadien (V–VI) definieren [24, 25]. Analog wurden von Thal 5 Phasen der Beta-Amyloid-Pathologie mit Ausbreitung von kortikal (Phase 1) über Hippocampus (Phase 2) und Stammganglien (Phase 3) in den Hirnstamm (Phase 4) und zuletzt in das Kleinhirn (Phase 5) definiert [26]. Zudem wurden die CERAD-Kriterien, mittels derer die Dichte der neuritischen Plaques in 3 Schweregraden (mild/moderat/schwer) wiedergegeben wird, festgelegt [27]. Die Kombination dieser Systeme wurde im *ABC*-System des NIA-AA (National Institute on Aging-Alzheimer’s Association, 2012) zusammengefasst, wobei A für die Beta-*A*myloid-Phase nach Thal, B für *B*raak-Stadium der NFT-Pathologie und C für den *C*ERAD-Score der neuritischen Plaques steht und jeweils mit einem Score von 0 (keine) bis 3 (schwer) bewertet wird [22]. Mithilfe dieses Scoringsystems wird neben der Schwere der Pathologie auch die Wahrscheinlichkeit angegeben, mit welcher diese Alzheimer-typischen neuropathologischen Veränderungen ein ausreichendes Substrat für eine Demenz sein können.

Interessant ist, dass die NFT Pathologie und die Beta-Amyloid-Pathologie zwar per se einem stereotypen stadienhaften Verlauf folgen, allerdings jeweils in unterschiedlichen Bereichen beginnen und in gegenläufigen Richtungen fortschreiten. Zumeist entsteht die Beta-Amyloid Pathologie noch vor der Tau-Pathologie, während die klinische Symptomatik der Alzheimer-Pathologie in erster Linie mit der Tau-Pathologie und weniger mit der Plaquepathologie zu korrelieren scheint [28].

Ein Ziel der Biomarkerforschung ist, neurodegenerative Erkrankungen bereits in einem frühen Stadium zuverlässig detektieren zu können und auch das Ausmaß der Erkrankung quantifizieren zu können. Hierfür stellen valide neuropathologische Stagingsysteme eine gute Grundlage dar. Einerseits können hier aus dem Liquor Gesamt- und hyperphosphoryliertes Tau sowie βA40-42-Werte gemessen werden. Zudem können in der Bildgebung Beta-Amyloid-Ablagerungen mittels spezifischer PET-Marker zuverlässig erfasst werden [29, 30]. Allerdings kann erst eine moderate bis häufige Dichte an neuritischen Plaques mittels Beta-Amyloid-PET detektiert werden [30]. Tau- und Mikroglia-PET-Untersuchungen werden derzeit noch primär im wissenschaftlichen Kontext angewendet [31].

Zudem werden neben zerebrovaskulären Veränderungen auch häufig verschiedene neurodegenerative Kopathologien beschrieben, die mit einem erhöhten Demenzrisiko bei vorliegenden Alzheimer-pathologischen Veränderungen verbunden sind [32]. Es ist beispielsweise in etwa 30–70 % der Alzheimer-Fälle auch eine TDP-43-Pathologie zu finden, überwiegend in Form der rezent beschriebenen „limbic-predominant age-related TDP-43 encephalopathy“ (LATE), die hauptsächlich Amygdala und Hippocampus befällt [33, 34]. Auch Lewy-Körper in der Amygdala wurden in bis zu 60 % der Alzheimer-Patienten beschrieben [35]. Solche Kopathologien müssen im klinisch diagnostischen und therapeutischen Setting bedacht werden.

## Zerebrale Amyloidangiopathie

Häufig mit Ablagerungen von Beta-Amyloid im Parenchym assoziiert ist die Ablagerung von Beta-Amyloid in Gefäßwänden, die zerebrale kongophile Amyloidangiopathie (CAA) bzw. Beta-Amyloid-Gefäßamyloidose, die mit einem erhöhten Risiko intrazerebraler, atypischer Blutungen einhergeht (Abb. 1g). Die Prävalenz der Amyloidangiopathie steigt mit dem höheren Alter, und das Risiko wird ebenso wie die Alzheimer-Pathologie durch einen APOE-E4-Träger Status erhöht [36]. Die Ablagerungen lassen sich wie Beta-Amyloid-Plaques im Gewebe mittels unterschiedlicher Techniken wie Kongorot-Färbung, Thioflavin S oder mittels Antikörper gegen Beta-Amyloid visualisieren. Bei der CAA kommt es überwiegend zu einer Ablagerung von Aβ40 mit einer Länge von 40 Aminosäuren [37]. Es können neuropathologisch verschiedene Typen der CAA unterschieden werden, z. B. Typ 1 mit Befall von Kapillaren und Typ 2 ohne Kapillarbefall [38]. Die Beta-Amyloid-Ablagerungen können die gesamte Gefäßwand oder auch nur einen Teil dieser betreffen und können zu Architekturstörungen wie Aufsplitterungen oder Mikroaneurysmabildungen der Gefäßwand führen, die letztlich deren Fragilität und die gesteigerte Blutungsgefahr erklärt [39]. Zudem kann die CAA im Rahmen einer sog. Amyloid-beta-assoziierten Angiitis (ABRA) mit einer oftmals granulomatösen Inflammation mit häufigen mit Beta-Amyloid-gefüllten bzw. phagozytierenden mehrkernigen Riesenzellen vergesellschaftet sein [40].

Ein interessanter Punkt, der derzeit diskutiert und beforscht wird, ist die potenzielle iatrogene Übertragbarkeit von Beta-Amyloid über chirurgische Eingriffe einschließlich Duratransplantaten mit Auslösen einer CAA [41].

### Hereditäre Formen

Neben der häufiger vorkommenden sporadischen Form der Alzheimer-Erkrankung sind auch genetische, autosomal-dominant vererbte Formen bekannt. Mutationen im *Amyloid Precursor Protein* (*APP, *Chromosom 21), *Presenilin‑1*(*PSEN1*, Chromosom 14)- und *Presenilin‑2*(*PSEN2*, Chromosom 1)-Gen wurden als Ursache der familiären Alzheimer-Erkrankung beschrieben [19]. De-novo-Mutationen in diesen Genen scheinen in Patienten mit früh einsetzender Alzheimer-Erkrankung allerdings auch nicht selten zu sein [42]. In der neuropathologischen Untersuchung kommen zumeist gleichartige Veränderungen wie bei der sporadischen Alzheimer-Erkrankung mit senilen Beta-Amyloid-Plaques und Tau-positiver Neurofibrillenpathologie zur Ansicht. Ein Teil der Fälle weist jedoch Veränderungen auf, die typisch für eine *PSEN1*-Mutation sind [43], wie z. B. sog. „cotton wool plaques“, die durch atypische, wie Baumwolle aussehende Plaques ohne dichten Beta-Amyloid-Kern im Zentrum geprägt sind. Besonders in *APP*-Mutationen wurde neben sehr großen Beta-Amyloid-Plaques im Parenchym zudem die Assoziation mit besonders ausgeprägter CAA beschrieben, die teilweise sogar die primäre Pathologie darstellt [44, 45].

### Down-Syndrom und Alzheimer

Eine potenzielle Verbindung zwischen Down-Syndrom und der Alzheimer-Erkrankung konnte aufgrund des deutlich erhöhten Risikos von Patienten mit Down-Syndrom, eine Alzheimer-Erkrankung zu entwickeln, und des üblicherweise deutlich früheren Beginns identifiziert werden [46]. Als Ursache dieses erhöhten Risikos wurde eine Überproduktion von Beta-Amyloid aufgrund des 3‑fach vorkommenden „Amyloid precursor protein“(*APP*)-Gens auf Chromosom 21 angenommen. Interessant ist hierbei jedoch, dass es nicht zu einer isolierten Ablagerung von Beta-Amyloid, sondern zu den typischen Alzheimer-Veränderungen mit Tau in Form von NFT kommt [47, 48]. Allerdings scheinen die Beta-Amyloid-Ablagerungen zuerst in Form diffuser Plaques vor der Tau-Pathologie aufzutreten und auch besonders ausgeprägt zu sein mit Ausbildung von größeren und komplexen Plaques. Die im Vergleich zur gesunden Population früher auftretende Demenz scheint jedoch primär mit dem Ausmaß der NFT-Pathologie zu korrelieren und nicht mit der Beta-Amyloid-Pathologie, wie es auch bei der sporadischen, adulten AD der Fall ist [49].

## Frontotemporale lobäre Degeneration (FTLD)

Die frontotemporalen Demenzen (FTD) wurden 1892 erstmals von Arnold Pick beschrieben und wurden gemäß ihrer klinischen Symptomatik als eine von der AD zu unterscheidende Erkrankung klassifiziert. So wurde diese neuropathologisch und auch klinisch heterogene Gruppe früher unter dem Namen „Pick-Demenzen“ zusammengefasst. Mit zunehmendem Verständnis der zugrunde liegenden Pathologien konnten jedoch unterschiedliche Erkrankungen identifiziert werden, die eine ähnliche Symptomatik induzieren. Häufig sind vergleichsweise junge Patienten (unter 65 Jahren) bereits betroffen. Klinisch können unterschiedliche Subtypen der FTD unterschieden werden. Neben der „Verhaltensvariante“ der FTD (bvFTD, vom Englischen „behavioural“) mit Verhaltensänderungen werden die primär progrediente Aphasie (PPA) mit prominenten Sprachstörungen und die häufig mit FTD assoziierten primär motorischen Erkrankungen (amyotrophe Lateralsklerose [ALS], kortikobasale Degeneration [CBD], progressive supranukleäre Paralyse/Blickparese [PSP]) unterschieden. Neuroanatomisch werden Frontal- und Temporallappen zu einem unterschiedlichen Ausmaß beeinträchtigt und letztlich atroph, daher die pathologische Bezeichnung FTLD („fronto-temporal lobar degeneration“) im Gegensatz zur klinischen Bezeichnung FTD („fronto-temporal dementia“). Häufig kommt es zudem auch zu einer Hippocampussklerose.

Als molekularpathologische Basis wurden bisher Tau‑, TDP-43 und FUS-Protein-Ablagerungen identifiziert, ein jetzt nur noch kleiner Teil der zugrunde liegenden Pathologien ist derzeit noch nicht identifiziert. Die Molekularpathologie zeigt hierbei eine Überlagerung mit dem FTLD-ALS-Spektrum.

### FTLD-Tau („Tauopathien“/Tau-Proteinopathien)

#### 3-repeat-Tau-Proteinopathien

##### Pick-Krankheit.

Diese 3R-Tau-Pathologie ist gekennzeichnet durch Ausbildung von sog. Pick-Körperchen. Diese Pick-Körperchen wurden erstmals 1911 von Alois Alzheimer beschrieben und später nach dem Erstbeschreiber des klinischen Bilds der frontotemporalen Demenz, Arnold Pick, benannt [50].

Neuropathologisch kommt es wie bei allen FTLDs zu einem schweren Nervenzellverlust in frontotemporalen Regionen mit oberflächlicher Spongiose (Abb. 1l). Oft finden sich auch ballonierte Zellen, welche auch als Pick-Zellen beschrieben wurden. Als distinktes histologisches Merkmal finden sich bei der Pick-Krankheit kugelförmige, bläulich tingierte (basophile), glatt umschriebene, argyrophile neuronale zytoplasmatische Inklusionen, bestehend aus 3R-Tau-Protein (Abb. 1h). Diese können bereits in der H&E-Färbung erkannt werden und mittels immunhistochemischer Färbungen für 3R-Tau bestätigt werden. Diese sind besonders gut im Bereich der Körnerzellen des Gyrus dentatus sichtbar. Die Pick-Körperchen finden sich jedoch auch im Hippocampus, in der frontalen, temporalen Großhirnrinde, im Cingulum und auch in subkortikalen Strukturen inklusive Basalganglien und Substantia nigra. Neben der neuronalen Pathologie kommt es auch zu oligodendroglialen und astroglialen Tau-Ablagerungen. Es wurden 4 sequenzielle Phasen beschrieben (I–IV) mit Beginn frontotemporal limbisch/paralimbisch (Phase I) und Ausbreitung in subkortikale Strukturen inklusive Stammganglien, Locus coeruleus, Raphe-Kerne (Phase II) mit anschließender Ausbreitung in die motorische Rinde und präzerebelläre Kerne (Phase III) und zuletzt Befall der visuellen Rinde (Phase IV) [51].

#### 4-repeat-Tau-Proteinopathien

##### PSP – progressive supranukleäre Paralyse/Blickparese.

Die PSP ist eine 4R-Tau-Proteinopathie, die klinisch eine Vielzahl an unterschiedlichen Subtypen aufweisen kann. Diese begründen sich Großteils auf den unterschiedlichen neuroanatomischen Verteilungsmustern der zugrunde liegenden Tau-Pathologie [52].

Neuropathologisch kommt es neben einem Neuronenverlust und einer Gliose in kortikalen und subkortikalen Kerngebieten zu einer Ablagerung von pathologischem Tau-Protein in Neuronen, Oligodendrozyten und auch Astrozyten (Abb. 1c). Die neuronale Tau-Pathologie besteht aus zumeist relativ großen globösen NFT, die oligodendroglialen Ablagerungen werden als „coiled bodies“ (CB) bezeichnet, und die astrozytäre Tau-Pathologie besteht aus sog. „tufted astrocytes“ (TA), auch als Astrozyten-Tangles bezeichnet. Die astrozytäre Tau-Pathologie ist zumeist in der grauen Substanz anzutreffen, die oligodendrogliale Pathologie ist in der grauen und auch in der weißen ZNS-Gewebesubstanz zu finden. Die Verteilungsmuster der Pathologien unterscheiden sich ebenfalls je nach Subtyp [53]. Üblicherweise überwiegt jedoch die subkortikale Pathologie mit einem deutlichen Befall der Stammganglien (v. a. Globus pallidus), Nucleus subthalamicus, des Hirnstamms (v. a. Substantia nigra, Colliculi, Locus coeruleus und Raphe-Kerne), geringer die unteren Oliven und der Zahnkern im Kleinhirn [54].

Auch für die PSP wurden neuropathologische Stadien vorgeschlagen. Ein Studie, welche die Ausbreitung der Pathologie in 3 verschiedenen PSP-Subtypen (Richardson-Syndrom, PSP-Parkinsonismus, PSP-Akinesie mit Gangblockaden) untersuchte, zeigte eine primäre Affektion von Nucleus subthalamicus, der Substantia nigra und des Globus pallidus und im Verlauf ein Ausbreiten in andere Regionen mit einer zunehmend höheren Gesamt-Tau-Last [55]. Rezent wurde zudem ein Stagingsystem mit 6 Stadien (1 bis 6) für den Richardson-Syndrom-Subtyp vorgeschlagen [54]. Es kommt hier in Stadium 1 zu einer neuronalen Tau-Pathologie in Nucleus subthalamicus, Globus pallidus und Substantia nigra und/oder zu einer oligodendroglialen Beteiligung im Globus pallidus und/oder zu einer astroglialen Beteiligung im Striatum. In Stadium 2 zeigen sich einzelne Tau-positive Zellen in der Frontalrinde und im Zahnkern. In Stadium 3 zeigen sich eine astrogliale Tau-Pathologie in der Frontalrinde und/oder eine neuronale Beteiligung im Zahnkern und/oder Tau-positive Oligodendrozyten im Kleinhirnmarklager. In Stadium 4 zeigt sich eine ähnliche Verteilung, jedoch ist die Menge an Tau-Pathologie höher. In Stadium 5 zeigt sich eine astrogliale Tau-Pathologie in der Okzipitalrinde, was in Stadium 6 an Menge zunimmt. Ein einheitliches Stagingsystem für alle Subtypen ist aufgrund der Diversität der Erkrankung derzeit nicht gut möglich.

##### CBD – kortikobasale Degeneration.

Eine weitere 4R-Tauopathie, die ebenfalls zu den atypischen Parkinson-Syndromen gezählt wird, ist die kortikobasale Degeneration. Neuropathologisch kommt es wie bei der PSP zu einer neuronalen, oligodendroglialen und astroglialen Tau-Pathologie ([56]; Abb. 1d) Die neuronale Tau-Pathologie in Großhirnrinde, Stammganglien und Hirnstamm besteht hier allerdings überwiegend aus Prä-Tangles in Nervenzellen. Die astrogliale Komponente zeigt sich in Form von sog. Astrozytenplaques (AP), v. a. in der Großhirnrinde. Hierbei weisen die Astrozyten im Gegensatz zu den „tufted astrocytes“ der PSP in den distalen Fortsätzen Ablagerungen auf, sodass diese eine plaqueartige Morphologie aufweisen. Die oligodendrogliale Pathologie besteht wie bei der PSP aus „coiled bodies“. Zudem sind ballonierte Neurone, ein Neuronenverlust sowie eine oberflächliche laminäre Spongiose in kortikalen Regionen zu finden, v. a. frontal und im Cingulum. Die astrogliale Pathologie stellt die früheste Läsion dar und kommt bereits in den präklinischen Phasen vor, progredient entsteht in Folge die neuronale Pathologie, welche im fortgeschrittenen Krankheitsstadium die gliale Pathologie überragt [57].

Das klinische Konzept des sog. kortikobasalen Syndroms (CBS) soll jedoch von der neuropathologischen Diagnose der kortikobasalen Degeneration (CBD) separiert werden, da diese nicht immer miteinander übereinstimmen und ein CBS auch auf Basis einer andersartigen Pathologie entstehen kann, u. a. einer PSP, einer Alzheimer-Krankheit oder einer FTLD-TDP (s. weiter unten).

##### AGD – „argyrophilic grain disease“.

Die AGD, auch als Silberkörnchenkrankheit bezeichnet, ist eine weitere, klinisch schwer zu definierende, 4R-Tauopathie, die neuropathologisch geprägt ist durch neuronale und gliale Tau-positive Inklusionen. Die neuronalen Inklusionen zeigen sich zumeist in Form von Prä-Tangles, und die glialen Inklusionen kommen einerseits als oligodendrogliale, kommaförmige zytoplasmatische „coiled bodies“ und auch als granuläre Inklusionen in Astrozyten zur Ansicht. Zudem zeigen sich typische körnige-Tau-positive, argyrophile (mit Silberfärbung anfärbbar) Strukturen, sog. „grains“, in neuronalen Dendriten ([58]; Abb. 1e) Neben den Tau-positiven Inklusionen sind auch immer wieder ballonierte Neurone im Mandelkern zu finden. Die Erkrankung involviert zumeist mesiotemporale und limbische Strukturen mit vorzugsweiser Involvierung des Gyrus ambiens, der Amygdala und des Hippocampus. Gemäß den sog. Saito-Kriterien können 3 Stadien der Erkrankung (I–III) unterschieden werden [58]. Aufgrund der lokalen Überlappung mit AD-Pathologie sowie der psychiatrischen Phänotypen bleibt die Erkrankung eine neuropathologische Diagnose.

##### GGT – „globular glial tauopathy“.

Die „globular glial tauopathies“ sind ein Spektrum von 4R-Tauopathien, bei denen es typischerweise zu runden oligodendroglialen und astroglialen Inklusionen mit schwerem Befall des Marklagers kommt ([59]; Abb. 1f). Es dominiert somit bei dieser Erkrankung die gliale Pathologie gegenüber der neuronalen. Die GGT wurde erst kürzlich als eigene Entität charakterisiert, und 3 verschiedene Subtypen (I–III) mit unterschiedlichen Verteilungsmustern und einer unterschiedlichen Involvierung von Oligodendrozyten und Astrozyten wurden definiert [59]. Typ I weist überwiegend globuläre oligodendrogliale Inklusionen (GOI), Typ II überwiegend eine Mischung aus GOI und „coiled bodies“ und Typ III überwiegend globuläre astrozytäre Inklusionen (GAI) auf. Klinisch zeigen sich entsprechend den unterschiedlichen Verteilungsmustern verschiedene Symptome, wobei Typ I primär eine frontotemporale Demenz, Typ II eine Motoneuronerkrankung und Typ III eine Kombination aus beiden induzieren kann. Nicht selten weisen die Patienten ein CBS oder einen PSP-Phänotyp auf. Zumeist tritt die Erkrankung sporadisch auf, es wurden jedoch auch genetische Fälle mit *MAPT*-Mutationen beschrieben [60, 61].

### FTLD-TDP

Eine weitere Form der frontotemporalen Demenzen ist jene, welche mit einer Ablagerung von TDP-43-Protein einhergeht. TDP-43 ist ein RNA-bindendes Protein, welches eine Molekülmasse von 43 kDa aufweist und in seiner physiologischen Form überwiegend im Kern der Nervenzelle vorkommt. Die genaue Funktion dieses Proteins ist noch nicht gänzlich geklärt, es dürfte jedoch eine wichtige Rolle im RNA-Metabolismus spielen [62]. Unter pathologischen Bedingungen wird TDP-43 hyperphosphoryliert und ubiquitiniert, und es kommt zu einer Verlagerung von nukleär nach zytoplasmatisch. Diese zuvor als FTLD‑U mit Ubiquitin-positiven Inklusionen bezeichnete Pathologie wurde 2006 erstmals in FTLD und bei der amyotrophen Lateralsklerose (ALS) bzw. Motoneuronerkrankung (MND) beschrieben [63]. FTLD-TDP und ALS scheinen 2 Enden des Spektrums dieser Erkrankung zu bilden. So finden sich neben reinen FTD-Phänotypen und reinen ALS-Phänotypen auch häufig MND-FTD- bzw. FTD-MND-Mischbilder. Die FTLD-TDP ist gekennzeichnet durch unterschiedliche, überwiegend neuronale TDP-43-Inklusionen (Abb. 1l). Neben kompakten neuronalen zytoplasmatischen Inklusionen (NCI) werden häufig auch dystrophe Neuriten (DN) und teilweise auch neuronale intranukleäre Inklusionen (NII) beschrieben. Daneben sind mithilfe der immunhistochemischen Färbungen gegen Phospho-TDP-43 auch granuläre und diffuse Inklusionen in Neuronen zu sehen (Abb. 1m). Gliale zytoplasmatische Inklusionen in Oligodendrozyten treten zumeist bei Motoneuronerkrankung auf. Anhand der Verteilungsmuster innerhalb der Rindenschichten (z. B. transkortikal oder in oberflächlichen Schichten) und Kombinationen dieser unterschiedlichen (kompakten oder diffusen) pathologischen Ablagerungen können 4 verschiedene Typen (A–D) unterschieden werden [64]. Diese 4 Typen scheinen unterschiedliche klinische Phänotypen und auch unterschiedliche genetische Assoziationen zu haben. Typ A mit TDP-43 Einschlüssen in oberen Rindenschichten geht primär mit einer bvFTD einher, Typ B mit transkortikaler und diffus-granulärer neuronaler TDP-43 Pathologie geht eher mit einer FTLD mit MND, Typ C, mit reichlich dicken Neuriten, liegt oftmals einer svPPA zugrunde, Typ D mit intranukleären TDP-43-Einschlüssen findet sich häufig bei Valosin-containing-protein(*VCP*)-Mutationen.

Das Verteilungsmuster ist überwiegend frontotemporal betont. Daneben sind zumeist der Hippocampus und subkortikale Regionen betroffen [64, 65]. Es kann allerdings auch zu einem deutlichen Befall von Stammganglien, Thalamus und Substantia nigra mit Ausbilden eines Parkinsonismus kommen. Im Kontext der bvFTD wurde auch hier ein sequenzielles Stagingsystem für die Auswertung der TDP-43-Pathologie entworfen [66]. Die 4 als „pattern I“ bis „pattern IV“ bezeichneten sequenziellen Stadien gehen von Amygdala und Gyrus rectus aus („pattern I“), breiten sich dann über die restliche Temporalrinde, das Cingulum und die Stammganglien aus („pattern II“), befallen in Folge die motorische Rinde, bulbäre somatomotorische Neuronen und das Vorderhorn des Rückenmarks („pattern III“) und zuletzt die visuelle Rinde („pattern IV“).

### FTLD-FET

Eine seltene Form der FTLD ist die FTLD-FET, bei der es zur Ablagerung von Proteinen der *FET*-Protein-Familie, eine Gruppe von RNA-bindenden Proteinen kommt. Zu dieser gehören „fused in sarcoma“ (*F*US), „Ewing’s sarcoma protein“ (*E*WS) und „TATA-binding associated factor 15“ (*T*AF15) [67]. Neuropathologisch kommt es zumeist zu neuronalen, seltener glialen, Inklusionen mit unterschiedlichen Verteilungsmustern (Abb. 1m). Es können neuropathologisch die 3 verschiedenen Formen „neuronale Intermediärfilament-Einschlusskörperchenkrankheit“ („neuronal intermediate filament inclusion disease“ [NIFID]), „basophile Einschlusskörperchenkrankheit“ („basophilic inclusion body disease“ [BIBD]) und „atypische FTLD-U“ (aFTLD-U) unterschieden werden.

Bei der **NIFID** zeigen sich neuronale zytoplasmatische sowie auch gliale zytoplasmatische Einschlüsse. Diese können mittels immunhistochemischer Färbungen gegen FET-Proteine markiert werden. Zudem weist ein Teil der Inklusionen auch eine Immunreaktivität für Intermediärfilamentmarker Internexin auf, was dieser Erkrankung den Namen verlieh [68]. Die Rolle, die diese Intermediärfilamente in der Erkrankung spielen, ist noch unklar. Die Erkrankung zeigt eine frontotemporal betonte Verteilung mit einer üblicherweise deutlichen Affektion des Caudatuskerns.

Auch bei der **BIBD** kommt es zu einer frontotemporal betonten Pathologie sowie einer Beteiligung des Nucleus caudatus und der Substantia nigra [69]. Selten ist sie Substrat einer juvenilen ALS. Es finden sich hier typische runde bläuliche bzw. basophile neuronale zytoplasmatische Inklusionen. Auch diese können mit FET-Immunhistochemie gefärbt werden [65].

Die **aFTLD‑U** führt neben einer frontotemporalen Atrophie zu einer schweren Degeneration des Caudatuskerns sowie zu einer Hippocampussklerose und geht mit neuronalen zytoplasmatischen und auch typischen filamentösen (vermiformen) intranukleären Inklusionen frontotemporal, im Hippocampus und im Striatum einher. Es können zudem auch hier gliale zytoplasmatische Inklusionen im Marklager vorkommen [65].

#### FTLD-UPS

Da mittlerweile die molekularbiologischen Grundlagen der meisten FTLD-Formen geklärt sind, ist die Gruppe der FTLDs, bei denen die zugrunde liegende pathologische Proteinablagerung unbekannt ist, klein. Diese Gruppe wird aufgrund der derzeit weiterhin nur mittels Ubiquitin detektierbaren Inklusionen derzeit noch als FTLD-UPS bezeichnet.

### Hereditäre Formen der FTLD

Häufig weisen FTD-Patienten eine positive Familienanamnese hinsichtlich demenzieller Erkrankungen oder Motoneuronerkrankungen auf. Hierbei sind beispielhaft Mutationen in den Genen für Mikrotubuli-assoziiertes Protein Tau (*MAPT*), die intronische Hexanucleotid-Expansions-Mutation im „Chromosome 9 open reading frame 72“(*C9orf72*)-Gen, Progranulin (*GRN*), „Valosin containing protein“ (*VCP*) und „charged multivesicular body protein 2b“ (*CHMP2B*) zu nennen ([70, 71]; s. auch Tab. 1).

**Table 1:** 

Gen	Chromosom	Pathologie	Häufige Klinik
*C9orf72*	9p21.2	TDP-43 + DPR	bvFTD, FTD-ALS, ALS
*GRN*	17q21.31	TDP-43	bvFTD
*MAPT*	17q21.31	Tau	bvFTD, PSP, CBS
*VCP*	9p13.3	TDP-43	FTD-ALS, bvFTD
*TARDBP*	1p36.21	TDP-43	bvFTD, nfvPPA, svPPA, FTD-ALS
*FUS*	16p11.2	FUS	ALS
*CHMP2B*	3p11.2	Ubiquitin, p62	bvFTD, FTD-ALS
*SQSTM1*	5q35.3	TDP-43	bvFTD, FTD-ALS
*TBK1*	12q14.2	TDP-43	bvFTD, FTD-ALS
*UBQLN2*	Xp11.21	Ubiquilin	bvFTD, FTD-ALS, PSP

*C9orf72* „chromosome 9 open reading frame 72“, *GRN* Progranulin, *MAPT* „microtubule associated protein tau“, *VCP* „Valosin containing protein“, *TARDBP* „TAR DNA binding protein“, *FUS* „fused in sarcoma“*, CHMP2B* „charged multivesicular body protein 2B“, *SQSTM1* Sequestosome 1, *TBK1* TANK-Binding Kinase 1, *UBQLN2* Ubiquilin‑2, *DPR* Dipeptid-Repeats

Die neuropathologischen Veränderungen können aufgrund ihrer Morphologie einen Hinweis auf die zugrunde liegende genetische Veränderung geben. So findet sich bei Progranulin-Mutation häufig eine Vielzahl von intranukleären, katzenaugenartigen TDP-43-positiven Inklusionen. Hexanucleotid-Expansions-Mutation im *C9orf72*–Gen führen zu charakteristischen kleinen neuronalen sternförmigen zytoplasmatischen p62-positiven Inklusionen, bestehend aus dem Expansionsprodukt in praktisch allen Hirnregionen. Besonders auffallend sind diese jedoch in den Körnerzellen des Gyrus dentatus des Hippocampus und der Kleinhirnrinde. *MAPT*-Mutationen induzieren eine ausgeprägte Tau-Pathologie in einem Ausmaß, welches das der sporadischen Tau-Proteinopathien übersteigt und teils für sporadische Formen atypische Merkmale aufweist.

## Synucleinopathien/Synuclein-Proteinopathien

Die Gruppe der Synucleinopathien stellt eine weitere Gruppe der neurodegenerativen Erkrankungen dar. In seiner physiologischen Form scheint das präsynaptisch vorkommende Alpha-Synuclein eine Rolle in der Synaptogenese und dem axonalen Vesikeltransport zu spielen. In seiner pathologischen Form kann es zu verschiedenen Erkrankungen kommen.

### Demenz mit Lewy-Körperchen.

Neben der Demenz vom Alzheimer-Typ und der Gruppe der frontotemporalen Demenzen ist die Lewy-Körperchen-Demenz (DLB) eine weitere häufige neurodegenerative Demenzursache.

Neuropathologisch kommt es zu einer progredienten Ablagerung von Alpha-Synuclein in Neuronen in Form von teils diffusen, teils gröberen und teils auch runden zytoplasmatischen Einschlüssen, die in der H&E-Färbung als eosinophile Lewy-Körperchen ersichtlich sind ([72]; Abb. 1j). Zudem können pathologische Alpha-Synuclein-Ablagerungen in Form von Lewy-Neuriten visualisiert werden. Diese Ablagerungen sind bei der DLB im Bereich der Großhirnrinde, in subkortikalen sowie Hirnstamm-Bereichen zu finden. Die Lewy-Körper im Hirnstamm weisen die klassische rundliche Morphologie auf mit einem dichten „core“ umgeben von einem helleren Halo, wohingegen die kortikalen Lewy-Körperchen in der H&E-Färbung viel schlechter definiert erscheinen. Das Verteilungsmuster der Pathologie kann anhand der sog. McKeith-Kriterien eingeordnet werden [72]. Es werden hier ein Hirnstamm – (Befall von dorsalem Vaguskern, Locus coeruleus, S. nigra), ein limbisches (Mandelkern, transentorhinale Rinde, Nucleus basalis Meynert, Cingulum) und ein neokortikales Stadium (Frontal‑, Temporal‑, Parietalrinde) unterschieden. Die neuropathologischen Kriterien wurden kürzlich aktualisiert [73]. Im Bereich der Großhirnrinde sind besonders in kleinen bis mittelgroßen Neuronen in tiefen Rindenschichten Lewy-Körperchen identifizierbar. In > 90 % der Fälle kommt es zudem zusätzlich zur Lewy-Körperchen-Pathologie auch zu einer Alzheimer-Pathologie mit Beta-Amyloid-Plaques und neurofibrillären Tangles [74]. Beta-Amyloid, Tau und Synuclein tragen hierbei allesamt zur kognitiven Dysfunktion bei.

### Morbus Parkinson.

Auch bei der Parkinson-Erkrankung kommt es im Verlauf oft zu einer demenziellen Entwicklung. Neuropathologisch finden sich wie bei der Demenz mit Lewy-Körperchen gleichartige Veränderungen mit runden eosinophilen neuronalen Inklusionen. Zu Beginn der Erkrankung kommt es primär zu einer Hirnstamm- und Riechsystem-betonten Pathologie, welche sich im Verlauf progredient in Richtung Großhirnrinde ausbreitet. Entsprechend wurden 6 Stadien definiert [75]. In den ersten beiden Stadien (1 und 2) kommt es aufgrund der Affektion von Bulbus olfactorius, dorsalem Vaguskern, Raphe-Kernen und Locus coeruleus zu einer entsprechend nichtmotorischen Symptomatik mit Riechstörungen, REM-Schlafstörungen, Depression und Obstipation. In Stadien 3 und 4 kommt es zu einer Affektion des limbischen Systems und des Meynert-Kerns sowie des Mittelhirns, insbesondere der Substantia nigra. Hier kommt es nach dem Verlust von 50–80 % der Neuronen zur der typischen motorischen Parkinson-Symptomatik. Im 5. und 6. Stadium kommt es schließlich zu einer Ausbreitung auf kortikale Bereiche.

Es ist weder klar, ob die Lewy-Körper per se das gesamte Substrat der kognitiven Einschränkung sind, noch ist gänzlich geklärt, inwieweit die Lewy-Körper im limbischen System bzw. Meynert-Kern sowie eine kortikale synaptische Dysfunktion die kognitive Symptomatik bedingen. Zudem scheint jedoch auch das Zusammenspiel mit Alzheimer bzw. Beta-Amyloid und Tau-Pathologie hier eine wichtige Rolle zu spielen.

Ein besonders interessanter Aspekt der Parkinson-Erkrankung ist der frühe und ausgedehnte Befall des peripheren autonomen Nervensystems durch die Synuclein-Pathologie [76]. Entsprechend kommt es zu einer Reihe von dysautonomen Symptomen wie etwa eine gastrointestinale oder urologische Dysfunktion sowie Blutdruckregulationsstörungen. Die verminderte autonome kardiale Innervation ermöglicht die Durchführung von (123)I-meta-iodobenzylguanidine(MIBG)-Szintigraphien zur Differenzierung von atypischen Parkinson-Syndromen [77]. Zudem ermöglicht der frühe Befall des peripheren autonomen Nervensystems den Nachweis von Synuclein in Biopsien (z. B. Haut, gastrointestinal, submandibulär), was eine potenzielle Frühdiagnostik erlauben würde [78]. Zudem begründet sich hierauf die Theorie, dass die pathologischen Synuclein-Ablagerungen primär peripher beginnen könnten und sich im Verlauf über den N. vagus in das ZNS ausbreiten könnten [79].

### MSA – Multisystematrophie.

Die Multisystematrophie ist eine weitere Synucleinopathie die als Hauptmerkmal gliale zytoplasmatische Inklusionen (GCI), sog. Papp-Lantos-Körperchen, in Oligodendrozyten, zumeist im Marklager, aufweist, die zu neuronaler Dysfunktion führen ([80]; Abb. 1k). Daneben sind auch neuronale zytoplasmatische und nukleäre Inklusionen zu finden. Es können die 2 klinikopathologischen Subtypen MSA-P/striatonigrale Degeneration und MSA-C/olivopontozerebelläre Atrophie unterschieden werden, die je nach Nervenzellverlust und Menge an GCI jeweils in 4 Schweregrade (0–III) eingeteilt werden können [81]. Die Kognition ist bei der MSA zumeist verhältnismäßig gut erhalten, allerdings kann es in einem Teil der Fälle zu einer demenziellen Entwicklung mit Einschränkung der Exekutivfunktionen und der verbalen Flüssigkeit bzw. Sprachflüssigkeit kommen [82]. Es wurde zudem rezent ein spezieller Subtyp der MSA mit Befall des limbischen Systems und der Großhirnrinde mit Entwicklung einer FTD als sog. FTLD-Synuclein-Subtyp beschrieben [83].

### Hereditäre Formen

Neben den üblicherweise sporadisch vorkommenden Synucleinopathien mit Parkinsonismus sind auch monogenetische Erkrankungen, die klinisch zu einem Parkinson-Syndrom führen, bekannt. Hierbei wurden beispielsweise Mutationen im *Synuclein*(*SNCA*)-, *Parkin*- oder *LRRK2*-Gen beschrieben [84]. Interessanterweise kommt es hier teilweise zu einer Diskordanz von Phänotyp und Genotyp. So zeigen etwa Mutationen im *Parkin-* und *LRRK2*-Gen zwar klinisch das Bild einer Parkinson-Erkrankung, neuropathologisch fällt hier jedoch das Fehlen von Lewy-Körper- bzw. Alpha-Synuclein-Pathologie auf [84]. Bei *SNCA*-Mutationen konnte zudem auch gezeigt werden, dass es hierbei zusätzlich zur Synuclein-Pathologie besonders häufig auch zu einer Tau-Pathologie kommt. Bei Patienten mit *SNCA*-Duplikationen und Triplikationen konnten interessanterweise auch Veränderungen ähnlich der MSA mit Synuclein-positiven GCI beschrieben werden [85]. Solche neuropathologischen Charakteristika müssen im Hinblick auf zukünftige zielgerichtete Therapien im Hinterkopf behalten werden.

## Trinukleotid-Repeat-Erkrankungen

Trinukleotid-Repeat-Erkrankungen entstehen auf Basis einer Anhäufung von Triplett-Repeats in bestimmten Genen, die eine Veränderung des entsprechenden Genprodukts führen. Im Rahmen der Vererbung über mehrere Generationen kann es durch Repeat-Instabilität zur Expansion der Triplett-Repeats kommen, was eine Antizipation der Symptomatik, also ein früheres Auftreten bzw. eine schwerere Erkrankung von Generation zu Generation, verursachen kann [86]. Erst ab einer gewissen Repeat-Anzahl kommt es zur entsprechenden Symptomatik, allerdings konnte für intermediäre Repeat-Anzahlen in unterschiedlichen Genen eine Beeinflussung des Phänotyps anderer Erkrankungen beschrieben werden. Beispielsweise wurde auf Basis von intermediären Repeat-Anzahlen im *ATXN2*-Gen eine Beeinflussung des Phänotyps von FTD mit einem gehäuften Auftreten von Parkinsonismus gezeigt [87].

Neben der Huntington-Krankheit sind viele weitere Trinukleotid-Repeat-Erkrankungen bekannt, die wie die spinozerebellären Ataxien, die Friedreich-Ataxie oder die myotone Dystrophie zu einer Vielzahl von neurologischen Symptomen führen können. Das Auftreten einer Demenz wurde zwar in einzelnen Fallberichten beschrieben [88, 89], scheint aber nicht die Regel zu sein.

### Huntington-Krankheit.

Die Huntington-Krankheit ist eine genetische, autosomal-dominant vererbte progressive neurodegenerative Erkrankung, welche durch eine Cytosin-Adenin-Guanin(CAG)-Trinukleotid-Expansion im *Huntingtin*-Gen auf Chromosom 4 verursacht wird [90]. Wenngleich die genauen pathophysiologischen Mechanismen der Erkrankung noch nicht gänzlich geklärt sind, scheint das mutierte Huntingtin-Protein eine toxische Wirkung auf Neurone, besonders der GABA-ergen „medium spiny neurons“ im Striatum zu haben, weshalb es zu einer progredienten Atrophie besonders im Bereich von Nucleus caudatus, Putamen und Pallidum kommt, welche sich im Krankheitsverlauf auf viele weitere Hirnregionen ausbreitet.

Mikroskopisch zeigen sich besonders im Striatum Veränderungen mit Neuronenverlust und einer Gliose. Mithilfe von immunhistochemischen Färbungen gegen mutiertes Huntingtin, Polyglutaminketten oder Ubiquitin können intranukleäre Inklusionen identifiziert werden. Der Schweregrad der Erkrankung wird mithilfe der Vonsattel-Kriterien in 4 Schweregrade (0 bis 4) je nach Ausmaß der Atrophie der Stammganglien eingeteilt [91].

### „Neuronal intranuclear hyaline inclusion disease“ – NIHID.

Die NIHID-Erkrankung ist eine unterdiagnostizierte progrediente neurodegenerative Erkrankung, die geprägt ist durch hyaline intranukleäre Inklusionen in Nervenzellen im Bereich des zentralen sowie des peripheren Nervensystems, Gliazellen und auch in diversen somatischen Zellen wie etwa Adipozyten im Bereich der Haut [92]. Die Inklusionen können mittels immunhistochemischer Färbungen gegen p62 und Ubiquitin als Autophagiemarker angefärbt werden. Als Substrat dieser Inklusionen wurde in einer asiatischen Studie in einem Teil der Fälle rezent das GGC-Trinukleotid-Repeat-Produkt in *NOTCH2NLC* beschrieben [93]. In der europäischen Population dürfte dieses Gen allerdings nicht ursächlich sein [94]. Die hier zugrunde liegende Mutation bzw. Mutationen sind derzeit noch unbekannt und Gegenstand intensiver Forschungsbemühungen.

Klinisch kann es zu verschiedenen Verlaufsformen kommen. Die adulten Formen sind oftmals geprägt von einer kognitiven Dysfunktion und einer typischen Leukenzephalopathie mit Signalanhebung im Bereich der Rinden-Mark-Grenze in der diffusionsgewichteten MRT [95]. Zudem kann es zu einer Bewegungsstörung und auch einer Muskelschwäche kommen. Aufgrund der weiten Verteilung der Pathologie besteht die Möglichkeit, die Erkrankung mittels Hautbiopsie zu diagnostizieren [92].

## Prionen

Prionenerkrankungen, übertragbare, stets letale neurodegenerative Erkrankungen mit unterschiedlichen Ätiologien (sporadisch, genetisch, übertragen) können klinisch mit einer Vielzahl an Symptomen bzw. beteiligten Systemen einhergehen. Auf die häufigste dieser Erkrankungen, die Creutzfeldt-Jakob-Krankheit, soll in der Folge kurz eingegangen werden. Im Vergleich zu anderen neurodegenerativen Erkrankungen sind Prionenerkrankungen zumeist durch einen rapiden klinischen Verlauf gekennzeichnet.

### Creutzfeldt-Jakob-Krankheit.

In ihrer klassischen Form ist die häufigste menschliche Prionenerkrankung, die Creutzfeldt-Jakob-Krankheit, geprägt von einer rasch progredienten demenziellen Entwicklung über zumeist wenige Monate, kombiniert mit weiteren betroffenen Domänen wie einer zerebellären Ataxie, einer Tonuserhöhung oder Sehstörungen [96]. Aufgrund ihrer Übertragbarkeit kommt den Prionenerkrankungen eine Sonderstellung zu. Im Gegensatz zu vielen anderen neurodegenerativen Erkrankungen existiert derzeit noch kein klassisches neuropathologisches Stagingsystem. Allerdings wurden diesbezüglich bereits Versuche unternommen, und es wird angenommen, dass sich die Prionpathologie von einem bestimmten Ausgangspunkt, evtl. vom Thalamus, in weitere Hirnregionen ausbreiten könnte [97].

Neuropathologisch kommt es bei Prionenerkrankungen zu spongiformen Veränderungen mit Ausbildung von Vakuolen im Neuropil, einer Astrogliose und markanter Mikrogliaaktivierung sowie einem Neuronenverlust (Abb. 1i). Mittels immunhistochemischer Färbungen und spezieller Gewebsvorbehandlungen können pathologische Prion-Protein-Ablagerung nachgewiesen werden. Je nach Subtyp können unterschiedliche morphologische und immunhistochemische Muster unterschieden werden. Diese diversen Subtypen setzen sich aus einer Kombination von dem Genotyp eines Polymorphismus auf Codon 129 des Prion-Protein-Gens (Methionin oder Valin; homozygot oder heterozygot) mit dem PrP-Typ im Westernblot (Typ 1 oder Typ 2) zusammen [96]. So können 6 (MM/MV1, MM/MV2, VV1, VV2) Subtypen unterschieden werden, welche sich sowohl pathologisch als auch klinisch im Sinne einer unterschiedlichen Erkrankungsdauer- und -symptomatik, unterschiedlicher Bildgebung und unterschiedlicher Ergebnisse der Liquoruntersuchungen anders verhalten. Es können jedoch auch Mischformen der Subtypen vorkommen und so ein komplexeres Bild bedingen [98]. Morphologisch können einerseits kleine und zarte in mittleren und tiefen Rindenschichten, andererseits aber auch große konfluierende Vakuolen in verschiedenen Regionen unterschieden werden. Zudem sind bei bestimmten Subtypen dichte unizentrische oder auch multizentrische PrP-Amyloidplaques zu erkennen. Immunhistochemisch kann unter anderem ein feines, diffus synaptisches Muster von groben Ablagerungerungen und Plaques oder plaqueartigen sowie perineuronalen Ablagerungen unterschieden werden. Die kortikalen spongiformen Veränderungen können nicht selten mithilfe von diffusionsgewichteten Sequenzen im MRT identifiziert werden. Zudem kann die Übertragbarkeit der PrP-Fehlkonformation verwendet werden, um mithilfe des sog. „real-time quaking induced conversion assays“ (RT-QuIC) konformationsabhängig pathologisches PrP im Gewebe oder Liquor detektieren zu können.

## Gemischte Pathologien

Das Vorkommen gemischter neurodegenerativer Pathologien ist häufiger als bisher angenommen [4] besonders in älteren Patienten. Besonders häufig kommen Alzheimer-typische Veränderungen in Kombination mit Lewy-Körperchen und/oder TDP-43-Protein-Ablagerungen vor [99]. Es wurde zudem postuliert, dass sich die unterschiedlichen Pathologien gegenseitig beeinflussen und weitere Neurodegeneration fördern können [100]. Dies muss natürlich auch bei der Interpretation von Biomarkern und bei der Therapieentwicklung bedacht werden.

## Sekundäre/primär nicht-neurodegenerative Demenzen

### Vaskuläre Demenz

Neben der Alzheimer-Demenz ist die vaskuläre Demenz eine der häufigsten Demenzursachen [101]. Hierbei können unterschiedliche vaskuläre Läsionen unterschieden werden. Neben der Multiinfarktdemenz mit multiplen kortikalen Infarkten können strategische Infarkte z. B. in Hippocampus oder Thalamus, die sog. Hypoperfusionsdemenz mit Grenzzonenverteilung, die hämorrhagische Demenz, beispielsweise bei CAA sowie Kleingefäßdemenz unterschieden werden. Eine Art solcher Kleingefäßerkrankung ist die subkortikale arteriosklerotische Enzephalopathie (Morbus Binswanger), bei welcher vaskuläre Läsionen primär im Bereich der Stammganglien und der weißen Substanz vorliegen.

Eine hereditäre Ursache der vaskulären Demenz stellt CADASIL dar („cerebral autosomal dominant arteriopathy with subcortical infarcts and leukoencephalopathy“), welche durch eine Mutation im *Notch3*-Gen bedingt ist und bei der es neuropathologisch zu einer Ablagerung von einem granulären osmiophilen Material in der Tunica media der Gefäßwände kommt [102]. Diese Veränderungen können elektronenmikroskopisch erkannt werden, aber auch indirekt färberisch in der PAS-Färbung, bei der die Gefäßwand einen granulären Zerfall aufweist. Eine spezifische immunhistochemische Färbung mit Antikörpern gegen die extrazelluläre Domäne von Notch‑3 kann weiterführend sein. Zudem kommt es zu den typischen neuropathologischen Veränderungen der Kleingefäßerkrankungen mit erweiterten Virchow-Robin-Räumen, Myelinabblassung und perivaskulären Pigmentablagerungen.

### Normaldruckhydrozephalus

Der Normaldruckhydrozephalus („normal pressure hydrocephalus“ [NPH]) ist in seiner typischen Form klinisch gekennzeichnet durch eine Kombination aus Demenz, Gangstörung und Harninkontinenz. Neuropathologisch können neben der Ventrikelerweiterung eine meningeale Fibrose, eine periventrikuläre ödematöse Myelinabblassung und Gliose sowie Ependymdisruptionen gezeigt werden [103]. Es konnte zudem gezeigt werden, dass NPH-Patienten gehäuft Alzheimer-Pathologie aufweisen [104].

### Posttraumatisch

Neben akuten und läsionellen traumatischen Veränderungen, welche selbstverständlich je nach Lokalisation eine entsprechende Symptomatik verursachen können, ist die chronisch traumatische Enzephalopathie (CTE) von steigendem Interesse.

Neuropathologisch zeigen sich typischerweise Ablagerungen von pathologischem hyperphosphoryliertem 3R- und 4R-Tau Protein in Nervenzellen (neurofibrilläre Tangles) und in Astrozyten, besonders perivaskulär und in den Tiefen der Sulci [105]. Zudem sind nicht selten TDP-43-immunreaktive neuronale und granuläre Inklusionen in Ammonshorn, Amygdala und anteromedialer Temporalrinde zu finden. Die genauen Mechanismen hinter der CTE sind derzeit noch ungeklärt. Es können entsprechend der topografischen Ausbreitung und der Schwere der Pathologie auch hier 4 Stadien (I–IV) unterschieden werden [106]. In Stadium I finden sich nur einzelne Tau-Ablagerungen in der Nähe von Gefäßen in der Tiefe der Sulci. In Stadium II sind bereits diskrete makroskopische Auffälligkeiten zu finden, und es kommt zu einer Ausbreitung der Pathologie sowie zu einer Abblassung der Substantia nigra. In Stadium III kommt es bereits zu einer deutlichen Atrophie und einer weiteren Ausbreitung der neuronalen und astrozytären Tau-Pathologie in kortikalen Regionen. In Stadium IV kommt es schließlich zu einer ausgeprägten Atrophie und ausgedehnten Tau-Pathologie.

### Demenz-Mimics

Besonders bei rasch progredienten demenziellen Prozessen bzw. auch bei Vorliegen von systemischen Symptomen oder auch bei einer ausgeprägten Bewusstseinsstörung sollte primär auch an das Vorliegen einer infektiösen oder inflammatorischen Erkrankung gedacht werden. Neben erregerdingten Enzephalitiden, die üblicherweise relativ rasch durch eine Lumbalpunktion ausgeschlossen oder nachgewiesen werden können, sind autoimmunologische Enzephalopathien von zunehmendem Interesse.

Bei Hinweisen auf eine Raumforderung als Grundlage einer demenziellen Entwicklung wird zumeist eine bioptische Untersuchung angestrebt. So kann zumeist relativ rasch eine entsprechende histologische Diagnose gesichert werden und eine passende Therapie eingeleitet werden. Allerdings gibt es neben den bereits in der Bildgebung klar als tumorverdächtig identifizierbaren Geschehen auch vereinzelt Malignome, welche weniger leicht als solche diagnostizierbar sind und entsprechend zu einer erschwerten Diagnose führen. Als Beispiel wären hier etwa intravaskuläre Lymphome zu nennen. Diese können klinisch, neben epileptischen Anfällen und Schlaganfall-ähnlichen Präsentation auch relativ häufig zu demenziellen Zustandsbildern führen [107]. Histologisch findet sich ein typisches Bild mit reichlich blastären, CD20-positiven B‑Zellen, die die Gefäße ausfüllen. Als weitere Beispiele seien die neoplastische Meningeose mit einem Tumorwachstum entlang der Meningen sowie gliomatöse Prozesse genannt. Auch hier kann durch eine entsprechende Biopsie zumeist zuverlässig eine Diagnose gesichert werden.

Zudem sollen auch andere behandelbare Erkrankungen wie toxische, metabolische und endokrinologische Ursachen für ein kognitives Defizit bedacht werden.

## Neuropathologie und Hirnalterung

Aufgrund des demografischen Wandels können zunehmend die Veränderungen in alternden Gehirnen untersucht werden. Neuropathologische Studien in besonders alten Personen sind von besonderem Interesse, um Veränderungen durch die natürliche Hirnalterung von neurodegenerativen Erkrankungen unterscheiden zu können. Die landläufige Meinung, dass mit steigendem Alter früher oder später jede Person eine Alzheimer-Erkrankung entwickelt, konnte so entschärft werden. Neurodegenerative Veränderungen stellen in jedem Alter einen nicht zwingend auftretenden, pathologischen Prozess dar.

Altern stellt jedoch einen bedeutsamen Faktor dar, der die Vulnerabilität für neurodegenerative Erkrankungen steigert. Dies beruht auf einer Vielzahl an Mechanismen, einschließlich eines Verlusts an Synapsen und Dendriten, was in Folge einen Volumenverlust bedingt [108]. Eine Rolle spielen hierbei beispielsweise dysfunktionale Mitochondrien [109], inflammatorische Prozesse [110], oxidativer Stress sowie eine gestörte Autophagie [111].

Den pathologischen Prozessen stellt sich die individuelle Reserve entgegen [112]. Hier spielen einerseits soziokulturelle Faktoren, Bildung und andererseits die Gehirnmasse bzw. die Menge an Synapsen und Dendriten eine Rolle [113]. Entsprechend konnte gezeigt werden, dass mithilfe von Lebensstilmodifikationen und durch Ausbau der kognitiven Reserve mithilfe von kognitiv stimulierenden Verhaltensweisen eine effektive Demenzprävention erreicht werden kann.

Letztlich sind jedoch die genauen Abläufe und Mechanismen der Hirnalterung und der Entwicklung eines kognitiven Defizits noch nicht gänzlich geklärt. Es ist daher die Forschung in diesem Bereich von fundamentaler Bedeutung um auf Basis dessen ein möglichst gesundes Altern ermöglichen zu können.
